# 437. A Single-Center Experience of Solid versus Hematologic Malignancies in People Living with HIV

**DOI:** 10.1093/ofid/ofac492.512

**Published:** 2022-12-15

**Authors:** Nour A Daoud, Mohammad Alfrad Nobel Bhuiyan, Satya Koppada, Mohammad Alam, Alexandre E Malek

**Affiliations:** LSU Health Science Center Shreveport, Shreveport, Louisiana; LSU Health Shreveport, Shreveport, Louisiana; LSU Health Science Center Shreveport, Shreveport, Louisiana; Louisiana State University Health Sciences Center, Shreveport, LA, USA, shreveport, Louisiana; LSU Health Shreveport, Shreveport, Louisiana

## Abstract

**Background:**

People living with HIV (PLWH) have an increased risk of cancer, including AIDS-defining and non-AIDS-defining malignancies. Some of the contributing factors are related to the high-risk lifestyle choices, but the introduction of HAART has reduced the incidence of AIDS-defining cancers.

**Methods:**

We conducted a retrospective study of PLWH who were diagnosed with cancer between November 2019 and March 2022. We identified 60 patients who had HIV infection prior to the diagnosis (dg) of cancer or concurrently diagnosed with cancer. We evaluated patient’s characteristics, CD4 T cell counts, HIV viral load, adherence to HAART, solid and hematologic (HM) malignancies.

**Results:**

Sixty pts were included in the analysis. The median age was 49.50 (range, 21-76 yrs) and 48 pts (80%) were male. The majority were African Americans (78.3%). Around 75% of pts were smokers, 35% of pts use alcohol, and 33.3% use illicit drugs. Cancer types were non-Hodgkin lymphoma (NHL) (11 pts, 18.3%), skin cancer (8 pts, 13.3%), Kaposi sarcoma (KS) (7 pts, 11.7%), prostate cancer (7 pts, 11.7%), head and neck cancer (5 pts, 8.3%), breast cancer (4 pts, 6.7%), Hodgkin lymphoma, leukemia, and lung cancer with same percentage each (3 pts, 5%), colorectal cancer (2 pts, 3.3%), and miscellaneous solid tumors (7 pts, 11.7%). AIDS-defining cancers were identified in 28.3% of pts. The median time between HIV and cancer dg was 8 years (range, 0-31 yrs). Eight pts (13.3%) were diagnosed with HIV and cancer at the same time [NHL (3 pts), myeloid leukemia (1 pt), and solid tumors different than cervical cancer (4 pts)]. A group of 43 pts (71.7%) had solid tumors were compared with 17 patients (28.3%) had HM. There were no differences between the two groups regarding gender, sexual orientation, smoking history, medical comorbidities, time between HIV and cancer dg. Pts with HM were younger (*P*-value of 0.05), and the rate of alcohol use was higher in pts with solid tumors (*P*-value, 0.003). HIV viral load > 200 copies/ml was higher in HM (75%) than solid tumors (44.1%) with *P*-value of 0.06 and the adherence rate to HAART was higher in pts with solid tumors (67.6%) compared to HM (38.4%) with a *P*-value of 0.1, prior to cancer dg.

**Conclusion:**

PLWH with HM are more likely to be younger, non-adherent to HAART, and have higher HIV viremia compared to pts with solid tumors.

**Disclosures:**

**All Authors**: No reported disclosures.

